# Lay beliefs about hypertension among HIV-infected adults in Kenya

**DOI:** 10.1136/openhrt-2016-000570

**Published:** 2017-03-01

**Authors:** Tecla M Temu, Ehete Bahiru, Fredrick Bukachi, Gerald S Bloomfield, Peter Muiruri, Carey Farquhar

**Affiliations:** 1 Comprehensive Care Center, Kenyatta National Hospital, Nairobi, Kenya; 2 Departments of Global Health, Medicine and Epidemiology, University of Washington, Seattle, Washington, USA; 3 Department of Physiology, University of Nairobi, Nairobi, Kenya; 4 Department of Medicine, Duke Clinical Research Institute and Duke Global Health Institute, Duke University, Durham, North Carolina, USA

**Keywords:** Explanatory, Model, HIV, Hypertension, sub-Saharan Africa, Cardiovascular diseases

## Abstract

**Objective:**

Hypertension affects 23% of Kenyans and is the most prevalent modifiable risk factor for cardiovascular disease. Despite this, hypertension awareness and treatment adherence is very low. We conducted a qualitative study to explore lay beliefs about hypertension among HIV-infected adults to inform the development of culture sensitive hypertension prevention and control program.

**Methods:**

Eight focus group discussions were held for 53 HIV-infected adults at the HIV clinic in Kenya.

**Results:**

Respondents had difficulties in describing hypertension. Hypertension was considered a temporary illness that is fatal and more serious than HIV. Stress was perceived as a main cause for hypertension with a large majority claiming stress reduction as the best treatment modality. Alcohol and tobacco use were not linked to hypertension. Obesity was cited as a cause of hypertension but weight control was not considered as a treatment modality even though the majority of our participants were overweight. Most participants did not believe hypertension could be prevented.

**Conclusion:**

Our findings suggest a limited understanding of hypertension among people living with HIV and points to an urgent need to integrate hypertension education programmes in HIV care facilities in Kenya. To effect change, these programmes will need to tie in the culture meaning of hypertension.

**Key questions**

**What is already known about this subject?**

Hypertension is the most prevalent modifiable risk factor for cardiovascular disease in Kenya.
Cultural contexts influence beliefs about hypertension.
Hypertension awareness and treatment adherence is low in both HIV-infected and HIV uninfected adults in Kenya.

**What does this study add?﻿﻿**

HIV-infected adults considered hypertension more serious than HIV.
Patients lay beliefs about hypertension causes, treatment and complications differed from the biomedical prospective.
There is an urgent need for integration of hypertension education programmes within the HIV care facilities in Kenya.

**How might this impact on clinical practise?**

The information obtained from this study can be used to design a culturally sensitive program to address hypertension prevention and management for people living with HIV.

## Introduction

Cardiovascular disease (CVD) is currently the third leading cause of death in Kenya, and the prevalence of CVD risk factors (CVDRFs) such as hypertension is significant.^1 2^ An estimated 40% of CVD-related deaths in low/middle-income countries occur in people <70 years of age, whereas only 11% of CVD-related deaths in Western countries occur in this age group.^3^ This combined pattern of high CVD burden at relatively younger age leads to large losses of potentially productive life-years. Hypertension remains the most preventable risk factor for CVD in Kenya with over 23.8% adults affected. Surprisingly, of all adults with hypertension only 22% are aware of their status and <3% of those individuals have their blood pressure under control.^2^

The scale-up of HIV treatment in sub-Saharan Africa has prolonged survival of people living with HIV (PLWH) who are increasingly at high risk for the CVD prevalent in their communities.^1^ PLWH in the developed countries are twice as likely to develop a stroke or myocardial infraction compared with their HIV-uninfected counterparts.^4–6^ There are ongoing efforts to understand the pathophysiology of HIV-associated CVD, and current theories suggest a multifactorial aetiology consisting of chronic inflammation, antiretroviral therapy (ART) and traditional CVDRFs.^6 7^ Among all modifiable CVDRFs, hypertension confers the highest attributable risk of CVD occurrence in PLWH.^6 7^ Despite multiple studies showing hypertension can be prevented or its onset delayed through lifestyle changes such as salt intake reduction, few hypertension prevention efforts have directly targeted this at-risk group. Given the high prevalence of hypertension in Kenya and this population at large, there is an urgent need to integrate hypertension prevention programmes within the existing and expanding HIV care infrastructure in Kenya that could be potentially accessible to the general population.^8 9^

To develop a successful programme that will assist patients to implement behaviour changes and properly manage their hypertension, healthcare providers need be aware of how cultural contexts influence beliefs about hypertension. Such knowledge facilitates effective health communication. The explanatory model (EM) provides a useful organising framework for eliciting this information.^10 11^ EM of an illness is an individual personal interpretation of causes, symptoms and treatment of that illness which is largely influenced by socioenvironmental factors. These lay beliefs may differ from the biomedical model. For example, Tanzanian hypertensive adults described hypertension as a transient condition that could be cured.^12^ Pork was cited as the main cause of hypertension in another study among African-Americans women in the USA.^13^ These beliefs are likely to affect health seeking behaviours and treatment compliance. To date, we are not aware of any studies that have assessed lay beliefs of hypertension in HIV-infected individuals. In this study, we report the EMs of hypertension among HIV-infected individuals in Kenya. This information will be used to design and plan a culturally sensitive programme to address hypertension prevention and management.

## Material and methods

This descriptive qualitative study was conducted at the Kenyatta National Referral and Teaching Hospital Comprehensive Care Center (CCC) in Nairobi County. Approximately 22, 000 PLWH receive care at the CCC with average daily attendance rate of 200 patients. Nairobi is the largest city in Kenya, located on the southern part of the country. Main economic activities are commerce, both large and small-scale and formal employment in the government or private sectors. The Kenyatta National Referral and Teaching Hospital CCC was chosen because it is located in the largest government referral hospital in Kenya and serves a diverse population of clients from and around Nairobi. It is therefore an ideal facility for recruiting patients with different demographic backgrounds. Data were collected through focus group discussions (FGD). FGD have been shown to provide broader insights on and better understanding of how people perceive a specific problem.

### Study participants

Participants were selected using a purposive sampling. Community health workers familiar with the clinic recruited study informants to capture variation in characteristics along the following dimension: gender and age. Each participant fulfilled the following inclusion criteria: (1) 18 years or older; (2) documented diagnosis of HIV; (3) fluent in English or Swahili. FGDs had an average of six participants (range 5–8).

### Focus group guide preparation and data collection procedure

The FGD guide was developed based on conceptual framework of the EMs (table 1).^11^ The guide was pretested in 10 in-depth interviews for its relevance and suitability. The principal investigator (TMT) and a research assistant (Hellen Okinyi) with appropriate language skills facilitated all FGDs. Interviews were conducted in English or Swahili lasting for 90 min. FGD were audiotaped with the approval of the participants. Interview transcripts were analysed serially and groups were added until the inductive analysis revealed no new concepts. At the end of the session, participants completed a demographic questionnaire and had their height, weight and blood pressure measurements taken as previously described.^14^

**Table 1:** Examples of questions used to elicit beliefs about hypertension

Causes	What do you think causes this problem hypertension?
Onset of symptoms	What happens to people when they get hypertension? What are the symptoms?
Pathophysiology	What does this illness do to a person? How does it work?
Course of illness	When people get hypertension what happens? How serious is hypertension? What are the problems this illness causes for a person?
Treatment	What can people do to take care of this illness? What kind of treatment should people receive from a doctor/nurse/clinic, if they have this illness? Can this illness be cured?
Prevention	Can you prevent hypertension? What can one do to prevent hypertension?

### Data analysis

Data included written notes and audio recording from the interviews. All recordings were transcribed verbatim. Translated scripts were read alongside original recordings to confirm accuracy. Written materials were imported into the Nvivo 11 software (QSR International) to facilitate coding. Using deductive reasoning results were grouped into predetermined categories based on the topic guide. Inductive reasoning was used to incorporate new and unexpected ideas. Coding was done by the principal investigator (TMT) and two other social scientists(James Ndimbii and Hellen Okonyi), after which all coded transcripts and thematic associations were crosschecked through discussions to resolve any discrepancies and reach consensus. Frequencies and percentages were calculated using SAS University Edition.

### Ethical consideration

Ethics review committees at the Kenyatta National Hospital and University of Washington approved this study. Each study participants received reimbursement to cover transportation costs.

## Results

Of the 53 participants, 58% were women. Groups at each meeting included individuals across the age range. Specifically, 23% of participants were 21–35 years of age, 54% were 36–50 years of age, 20% were 51–65 years of age and 3% were above 66 years of age. Participants were well educated, with 45% having at least some postsecondary education. Eleven participants (20.6%) were self-reported hypertensives. Of the 11, 9 reported to have been prescribed hypertension medicines but only 2 reported to have been taking their medications as prescribed at the time of recruitment. Other baseline characteristics of the group are shown in table 2.

**Table 2:** Basic characteristics of study participants

Characteristics	n = 53
Female	31 (58.4)
Overall age data (years) 18–35 36–50 51–70	12 (23) 29 (54) 12 (23)
Education Tertiary (>12) Secondary (9–12 grade) Primary (0–8 grade) Never attended school	24 (45.2) 18 (33.9) 10 (18.8) 1 (1.8)
Marital status Single Married Widowed Separated	15 (30.3) 27 (50.9) 4 (8.4) 5 (10.4)
Current professional status Employed Self-employed Unemployed	26 (49) 23 (43) 4 (8)
Medical history High Cholesterol Diabetes High blood pressure Smokers Alcohol users	2 (3.8) 3 (5.6) 11 (20.6) 12 (22.6) 12 (22.6)
HIV-related characteristics	
Median HIV infection duration (years), IQR	7 (12)
ART naive	6 (12.2)
BMI, kg/m^2^, SD	26.5±6.0

Values are n (%), median (IQR) or mean±SD.

ART, antiretroviral therapy; BMI, body mass index.

### Understanding of high blood pressure

Hypertension was commonly referred as ‘pressure’, and all participants were aware of this term. When participants were asked what they understand by the term pressure, majority were reluctant to answer and admitted they did not know enough to answer this question. They describe hypertension around alteration of blood flow describing it as, ‘rushing of blood through the veins faster than it should.*’* A 46-year-old female respondent said, “I think it happens to people many times … at least everybody experiences that pressure … you can’t be normal at all times because it comes with the issues of getting angry … getting shocked.”

Respondents were unable to provide correct normal blood pressure readings but were interested in learning this information. A 56-year-old female with a 10-year history of hypertension said, “Many times I ask the nurse and I am told your blood pressure is okay … sometimes it is 80/60 … 100/80 so I don’t know exactly which is which but I will like to know*.”*

### Causes

There were a number of factors that were perceived to cause hypertension. The most frequently mentioned cause was stress. Others included hereditary, excessive salt intake, antiviral drugs and hot pepper (table 3).

**Table 3:** Patient perspectives on hypertension: causes, complications and treatment

**Perceived causes**	**No of FGD**	**Perceived complications**	**No of FGD**	**Perceived treatment**	**No of FGD**
Stress	8	Sudden death	5	Stress reduction	6
Hereditary	4	Headache	1	Medication	3
Salt	2	Poor vision	2	Salt reduction	3
Fatty foods	2	Heart disease	3	Milk	1
Arthritis medications	1	Kidney diseases	1	Banana	1
Hot pepper	1	Stroke	2	Exercise	1
Red meat	1	Emotional disturbance	1	Cut fat foods	1
Coffee	1	Liver disease	1	Water	1
Viagra	1	Pancreatic diseases	1		
Antiretroviral drugs	1	Nose bleed	1		
Arthritis medications	1				
Obesity	1				

FGD, focus group discussion.

#### Stress

Majority of the participants reported that management of life stressors was a key determinant of whether one would develop hypertension or not. Stressors were described as issues that resulted from thinking and worrying too much, tension, anxiety, anger and disagreements. A number of participants discussed how they or a family managed pressure after experiencing these stressors. A 36-year-old female stated, “Blood pressure is caused by stress … my mum has pressure because she used to worry a lot about my brother who drink too much …. She takes medications daily …. She has to take medication to control otherwise if she doesn’t use it … she will be complaining of dizziness and headache.”

Several hypertensive respondents believed that worrying too much about their HIV diagnosis also caused hypertension. A 42-year-old respondent with a 4-year history of hypertension stated, *“*My pressure was caused by thinking too much … being in denial and refusing to accept my status. When I was diagnosed of HIV …. I used to think I would not see another day …. I believe that is what caused me to have pressure.”

#### Hereditary

Even though most participants believed that stress is the main cause of hypertension, a number of participants (particularly the hypertensive informants and those with a family member with hypertension) believed that hypertension could be passed down from a relative. A 52-year-old hypertensive respondent said, **“**Like in my case …. I have not experienced something bad to cause me stress …. I think my mother passed it to me she had pressure*.”*

Another 41-year-old hypertensive female responded*, *“**Pressure can be hereditary … like in our family my mum has pressure … my sister has pressure okay I have refused mine … it is there but I have refused it … to clear it you either use drugs or your will power … allow yourself to control it … like my sister and mom take medications daily …. I have refused to take drugs completely … so for me I believe it can clear, it all depends with how you handle issues.”

#### Lifestyle behaviours

Few respondents considered diets rich in fats, uncooked salt and physical inactivity as possible causes of hypertension. A 47-year-old hypertensive female responded, **“**Salt that is not cooked can give you pressure.”

A 52-year-old hypertensive respondent stated, **“**Pressure is caused by our lifestyle … what we eat … such like things contribute a lot in blocking the veins, and after blocking the veins you will automatically get that disease.” A number of participants associated hypertension with 'big people', a belief based largely on what they had observed in family members or associates. Others claimed non-adherence to ARV can also result in pressure. A 38-year-old male said, **“**Even with these drugs that we are using, failing to adhere to the timings like if its 8 it should be 8 can cause pressure*.”*

None of the participants mentioned tobacco, age or alcohol even though over 20% of the interviewers were either smoking or alcohol users.

### Pathophysiology

When asked about how the causes mentioned produce hypertension, participants had very little to say. Some informants indicated that stress increases blood flow leading to strain on the heart. A 41-year-old female stated, **“**If you have pressure, the blood flows very fast in the heart … and you get the heart attack.”

Others attempted to describe the complications based on what they believed was the pathophysiology of hypertension, as illustrated by a 65-year-old hypertensive male, *“*When the pressure is high … you could get a blood clot which could lead to stroke depending on where the blood clot is … maybe it could be on the leg*.”*

### Course of illness

Participants considered hypertension fatal. They perceived hypertension as a more serious illness than HIV. A 57-year-old female with a 8-year history of hypertension stated, **“**This sickness according to how I know it and how we have been explained to by doctors … is bad than HIV because … you cannot die from HIV if you are taking medication … but pressure you can be on medication but you encounter something that shock you … and may go to sleep and never wake up in the morning.”

There was universal belief that hypertension was a temporary and symptomatic condition. Hypertensive participants claimed that listening to their bodies was sufficient to tell whether their pressure was high. Headache and dizziness were cited as important indicators for high blood pressure. Hypertensive respondents interpreted the meaning of the symptoms experienced and adjusted their lifestyles and treatments accordingly. A 42-year-old female hypertensive summed it all, “Pressure has signs …. I normally know when my pressure is high cause I get migraine on the forehead or I see black … when you see black then you know pressure is abnormal … personally …. I refused medication because there is no way I can take the medication for the virus and then add for pressure … that is too much drugs in your body. I told the doctor …. I will control my pressure because there is a way you can feel it is high.”

Informants reported that hypertension could have immediate effects on your body such as sudden death, paralysis, loss of sight and heart attack but were oblivious of the chronic nature of hypertension. Kidney diseases were rarely mentioned as a complication of hypertension (table 3).

### Treatment

All focus groups participants had some knowledge regarding of treatment of hypertension. Treatment modalities were grouped into medical and non-medical strategies (table 3). Stress reduction and lifestyle changes were the most frequently cited treatment strategies. A 29-year-old male stated, **“**Dad does not prefer stress …. That is why we are staying with him … my mom died and I live with my uncle and two children … we are the only people around him and ehh the other drunkard uncles are away in Nairobi … he requests us to talk to them since if his pressure will go up he will die … what my dad tells us is that he doesn’t want to be annoyed because if he does … the heart might stop functioning and he will die.”

Lifestyle changes referred by the respondents included exercising, proper sleep, drinking a lot of water and proper diet with less fat and salt. Several hypertensive respondents emphasised the importance of medication. A 48-year-old female with a 5-year history of hypertension stated, “You cannot survive without medication … if we have to take a lot of medication, we take a lot of water so that we are able to flush those drugs from the blood stream, so you can’t avoid medication when there is pressure.”

Few informants cited conventional medicine with herbal remedies such as tea and garlic.

Deterrents to taking prescribed medications included drugs side effects, cost, availability and general lack of knowledge about the illness. A 58-year-old hypertensive male said, *“*My doctor asked me if I have been taking pressure drugs and I say no… so he then advises me to go buy and use the drugs he had prescribed until the next visit …. When I went home, I figured …. I am taking too much drugs. The ones for HIV and pressure and I decide to try exercise instead*.”*

Another hypertensive male responded, “It is very tough as a bus driver whenever I take the pressure pill I have to go to the toilet all the time so …. I chose to only take it when I feel sick*.*”**

The cost of these medications was also a concern. A 58-year-old participant explains, “I have been prescribed two medications a day but I usually take one a day because I can’t afford and sometimes none unless I have a headache.*”*

### Prevention of hypertension

Participants discussed different ways of preventing hypertension. Most participants believed that there was little one could do to prevent hypertension partially because some life stressors are unavoidable while few reported that exercising and dietary changes such as cutting down on fried food and salt could prevent hypertension. Weight control and smoking cessation cited as modalities for hypertension prevention.

## Discussion

Eliciting PLWH understanding about hypertension is crucial because it increases the awareness of cultural factors that often are not well understood by health professionals responsible for treatment and prevention of hypertension. To our knowledge, this is the first study to explore EMs of aetiology, course of illness and treatment of hypertension in this at-risk population. Respondents had limited information about hypertension; however, they believed strongly that it is a serious condition that could lead to fatal outcomes. They perceived it as a more serious illness than HIV. They also mention they had little information about hypertension prevention. Since the most effective way to control hypertension is prevention, this finding particularly troublesome. Moreover, the belief that there is little one can do to prevent hypertension and that it leads to an early death makes it clear that much more hypertension information should be disseminated to PLWH. Similar research is needed in PLWH to compare these findings.

With regard to the causes, many participants expressed uncertainty while those certain cited stress as the main cause of hypertension. This view has been found in similar studies of patients with hypertension and is likely to underscore the need of pharmacotherapy treatment.^12–17^ In spite of this, none of the participants could cite a stress reduction approach for hypertension. There is increasing evidence suggesting the contribution of psychosocial stress in the development of hypertension;^18 19^ however, stress reduction therapy alone has not been effective in the control of hypertension.^20^ Thus, patients need to be educated on the multifaceted approach needed for the treatment of hypertension.

Another finding that is of concern is that none of the participants saw a link between alcohol or tobacco and hypertension even though both risk factors were prevalent in this group. A potential explanation for our observations is that in the country like Kenya where there is an acute shortage of workforce, physicians may not have useful time to talk about hypertension and may still regard CVD risk to be low in this population.^14^ Further studies are needed to determine if the poor perception about the risk factors of hypertension is related to the failure of clinicians to educate patients or patient simply misunderstandings.

Obesity was believed to cause for hypertension however none of the participants cited weight control as treatment despite their mean body mas index being in the overweight range. Body size misperception, a failure to recognise the need to lose weight, has been identified as an obstacle to weight reduction and as a target for intervention for hypertension risk reduction among patients with hypertension in SSA.^12^ Our results demonstrate that participants did not have a real view of their own susceptibility. Therefore, they did not see the need to lose weight. Interventions targeting overweight/obese patients on hypertension risks are needed.

The misconception that hypertension is an episodic illness associated with symptoms (particularly headaches) is a belief that discounts hypertension as a chronic disease. These findings are consistent with data on ethnic minority patients with hypertension elsewhere.^12 16^ Clearly, participant’s belief and the biomedical belief are in conflict. Maintaining different understanding of hypertension follows that physician and patients envision different ways of treating hypertension. Likewise, even after patients have received care, treatment adherence may be compromised if patient’s expectation is just symptom relief.

Despite the fact that hypertension was considered fatal, participants did not seem to recognise the risks associated with hypertension drugs non-adherence. For example, all participants reported to have been taking ARVs but only two out of nine hypertensive reported taking their hypertension drugs as prescribed. Our results have important clinical implication; HIV clinicians should not assume that adherence is consistent across comorbid conditions in the same individual. According to Leventhal’s Common Sense Mode of self-regulation, medication adherence is driven by patients’ conceptualisations of their illness.^21^ In this study, the lack of recognition of the silent chronic nature of hypertension may have resulted in reluctance to adhere to hypertension medication. Similar campaigns used to advocate ART adherence in these clinics should be adapted for hypertension drug adherence.

Participants had limited information on hypertension prevention, majority were unsure expressing that hypertension may be inevitable, even when one tries to prevent it while others cited the hereditary nature of the disease. All focused group participants recruited for the study were regularly screened for hypertension at the HIV clinic.

### Limitations

The study had some limitations. It was a small qualitative study of participants actively engaging in HIV care, which we would have benefited from talking with PLWH who are not currently in care. As such the results may not be generalisable to the larger HIV-infected population. We however need to point out that identifying HIV-infected individuals and obtaining the kind of information we desired outside the HIV clinics is very difficult.

## Conclusion

Our findings suggest a limited understanding of hypertension causes, treatment and prevention among PLWH in Kenya and points to an urgent need for integration of hypertension education programmes within the HIV care facilities in Kenya.

Understanding culture-based beliefs is critical for design of effective and successful programme since these lay beliefs are likely to determine patient’s perception of risk to develop hypertension and decision to accept treatment. Our results provide insights into the design of future intervention programmes aimed at hypertension prevention and control in this population.
